# Involvement and Autonomy of Minors in Medical Settings: Perceptions of Children Undergoing Surgery and Parents

**DOI:** 10.3390/children10121844

**Published:** 2023-11-24

**Authors:** Francisco J. Rodríguez-Domínguez, Teresa Osuna-García, Alberto Guillén, María D. Pérez-Cárceles, Eduardo Osuna

**Affiliations:** 1 Service of Otorhinolaryngology, University Hospital of Cartagena (Murcia), 30202 Murcia, Spain; franciscoj.rpdriguez6@carm.es (F.J.R.-D.); albertoj.guillen@carm.es (A.G.); 2 Service of Pediatrics, University Hospital of Caravaca de la Cruz (Murcia), 30400 Murcia, Spain; teresa.osuna@um.es; 3 Department of Legal and Forensic Medicine, University of Murcia, 30120 Murcia, Spain; mdperez@um.es

**Keywords:** informed consent, competence, health decision, children, parents, ethics

## Abstract

Informed consent presupposes competence and represents a formal decision by an informed person who has the legal capacity to accept medical action or participate in research. Our aim was to analyze the perceptions of minors and their parents about the age at which they consider that a minor is competent for making health decisions. A descriptive observational study was carried out in 302 minors between 12 and 17 years of age undergoing elective surgery, and 302 parents (range 30 to 62 years). Two semistructured questionnaires were designed, one for the minors and the other, for the parents. A total of 20.1% of minors and 31.1% of parents believe that patients should not make decisions related to their health until they are 18 years old. A total of 74.9% of the minors surveyed consider that from 16 years of age, the minor is empowered to make decisions. In parents, this percentage is 60%. In the pediatric setting, each case and situation must be examined individually to determine if the minor meets the condition of maturity to decide. The ideal is to promote the minor’s participation in decision-making, giving them the opportunity to participate in the process in a manner appropriate to their capacity.

## 1. Introduction

Respect for the will of children is highlighted in Article 12 of the United Nations Convention on the Rights of the Child [1]. This agreement recognizes children as rights holders, draws attention to their protection and provision of rights, the obligation to consider their best interests and evolving capacities to make sound decisions and participate in promoting their own welfare. Decision-making capacity and legal competence are terms often used interchangeably in a hospital or health setting to describe an individual’s ability to make thoughtful decisions related to their health and acceptance or refusal of treatment, as a manifestation of their autonomy. The ability to make decisions can be defined by four standards: expressing a choice, understanding, reasoning, and appreciation [2,3]. To be competent enough, it is necessary that the subject has the mental capacity to make decisions, but they must also be responsible for the decision in the specific situation. 

In pediatric settings, the decision-making process for medical treatment and participation in research is not fully clarified, and terms such as consent and assent are frequently used, which for some authors sometimes causes confusion [4,5]. Informed consent presupposes competence and represents a formal decision by an informed person who has the legal capacity to accept medical action or participate in research. However, assent implies agreement by a person who does not have the legal capacity to consent and therefore requires the consent of a legally authorized person (in the case of a minor, the parents or legal guardians). In emergency departments, legal standards and medical ethics recommendations support treatment regardless of consent issues. Appropriate medical care for the pediatric patient with an urgent or emergent condition should never be denied or delayed because of problems obtaining consent [6]. 

Respect for the wishes and preferences of a child in their healthcare decisions recognize that they have a voice which is important and worth hearing [7]. Children who lack the decision-making capacity have preferences and opinions about their medical care, even if they do not have the last word on it. According to the English Gillick law, a competent child (or in the US a mature minor) ‘achieves a sufficient understanding and intelligence to enable him or her to understand fully what is proposed’ and ‘sufficient discretion to enable him or her to make a wise choice in his or her best interests’ [8]. Competence has often been associated with age, cognitive ability, and rationality [9]. However, there is a lack of harmonisation as regards the age of the mature minor to consent, and there are no systematic tools available to assess competence in decision-making capacity [10]. In some countries, minors are allowed to make healthcare decisions from a fixed age below the legal majority. In Spain, as in many other countries, it is considered that at 16 years of age, a patient possesses competence for medical decision-making unless it is proven otherwise.

In addition to age, as an objective criterion to consider a minor as autonomous to consent to medical treatment, a subjective component is also included in those situations in which children are capable of understanding the proposed medical intervention, regardless of their age [11,12,13]. In Spanish legislation, as in many countries, when caring for patients under 16 years of age, doctors can accept the minor’s consent if they are considered to have sufficient competence to fully understand what is being proposed to them. This subjective criterion is more difficult to establish because the development process of any minor will depend on personal, family, and cultural experiences, and will not be the same in all cases. Furthermore, decision-making competence is not an intermittent phenomenon, but depends on the medical intervention and the specific situation [14]. In those cases where there is insufficient competence to give informed consent, parental permission is required and the best interests of the patient must be addressed. In the event of a discrepancy about what the parents or the doctors consider to be the best interests of the minor, judicial intervention must be requested, except in emergency conditions in which the doctor can adopt the necessary measures to safeguard the life and health of the patient.

The aim of this study is to analyze the perceptions of minors who have undergone surgery, comparing them with the perception of their parents about the degree of understanding of the information received, the process of obtaining informed consent, and the age at which they consider that a minor is competent to make medical decisions in clinical interventions of varying severity. To carry out this study, we have given, as examples, different situations in which a minor can be treated by a doctor.

## 2. Materials and Methods

This study was carried out in a University Hospital (University of Murcia, Spain) for two months (September and October 2022). A descriptive observational study was carried out in minors between 12 and 17 years of age undergoing elective surgery. One of their parents (father or mother) who accompanied the child or adolescent during their hospital stay and who received information about the surgical intervention was also included in this study. All participants provided informed consent to participate, and this study was conducted in accordance with the approved University of Murcia Research Ethics Committee protocol (approval no. ID 2016/2018). 

Two semistructured questionnaires were designed and implemented, one for the minors (15 questions) and the other, for the parents (20 questions), to analyze perceptions about the medical decision-making process involving minors, as well as who should give their consent. The questionnaires focused on the explanation provided by the doctor, the understanding, and the provision of consent in the surgical intervention and other possible situations. The questions were written in understandable language. The questionnaires include closed and open questions and a prior validation period. A questionnaire was designed that served as a pretest, through which 30 patients and one of their parents (father or mother) were interviewed with the objective of knowing the need to modify, add, or eliminate questions and detect errors or limitations of the interview. Once reviewed, it was administered to a new sample of 20 patients and their parents, selected through consecutive sampling to obtain the definitive questionnaire. These questionnaires were carried out during the months of June and July 2022, and were discarded for the study. Except for cognitive problems or language difficulties due to language comprehension in foreign subjects, we did not find difficulties in its administration.

The questionnaires were answered individually, in the hospital, one week after the surgical intervention. Participants were assured of the confidentiality of their answers both with respect to third parties and other participants (children/parents). The average time to answer the questionnaire was 20 min for minors and 25 min for parents. 

The data were processed using the statistical package SPSS v.24.0 (IBM SPSS Statistics, Inc., Chicago, IL, USA), for Windows. Descriptive statistics were applied. Pearson’s coefficient and Fisher’s exact test were used to identifying the degree of correlation or association between variables. A value of *p* ≤ 0.05 was considered be statistically significant. Cohen’s kappa index (k) was applied to give a quantitative measure of inter-rater agreement for the minors’ responses and those of their parents in similar questions. The k value was ranked, according to Landis and Kock [15], as poor (0–0.2), fair (0.21–0.4), moderate (0.41–0.6), substantial (0.61–0.8), or almost perfect (>0.8).

## 3. Results

### 3.1. Participants

**Figure 1:**
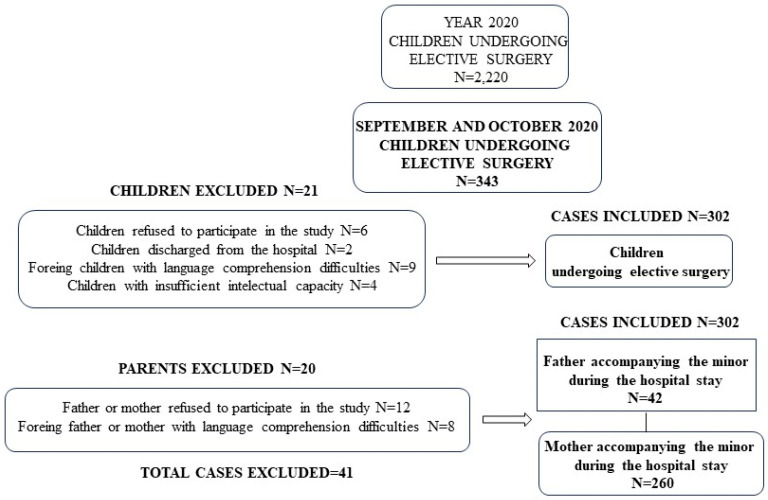
Diagram for the selection of participants in this study and causes of exclusion.

The total number of children between 12- and 17-years old undergoing elective surgery during 2022 was 2220, and during the two months of the study (September and October), it was 343. In 41 cases, the patients and/or the parents refused to take part in the study, or it was impossible to conduct the interview because the patient had been discharged from the hospital, or their insufficient intellectual capacity, or due to language-related problems (Figure 1). 

Our study comprised 302 minors (47% males and 53% females) with a mean age of 15.0 ± 1.8 years (mean SD; range 12 to 17 years), and 302 parents (father or mother) with an age of 43.1 ± 6.3 years (mean SD; range 30 to 62 years). Minor and parent demographic characteristics are shown in Table 1.

In relation to the distribution of minors according to age, 49.0% were aged 16 or 17, and 51.0% were less than 16 years old. The physician in charge of the patient was asked about the severity of the surgical intervention. In 135 (44.7%), the interventions were considered by the doctor as serious. In 260 cases (86.1%), the parent’s questionnaire was answered by the mother, and 42 questionnaires (13.9%) were answered by the father. 

### 3.2. Information Provided by the Doctor

A total of 82.5% of the children (N = 249) responded that they received information about the surgical procedure to which they were going to undergo, and 73.5% (N = 222) understood the information provided. A total of 17.5% (N = 53) of the minors did not receive information about the surgical procedure to which they were going to undergo. In 5 of these cases, the intervention was considered serious by the doctor, in 31 cases the minors were under 14 years of age, and in 22 cases they were over 14 years of age (Table 2). 

In the group of parents, in 82.7% (N = 250), they answer that the doctor has explained the pathology and the surgical intervention performed. Only 19 subjects (6.3% of those who were informed) did not understand it. The content of these responses was contrasted with the diagnosis and treatment recorded in the medical history to check whether they truly knew the pathology that the child presented. 

### 3.3. Age Maturity in Health-Related Decisions

**Figure 2:**
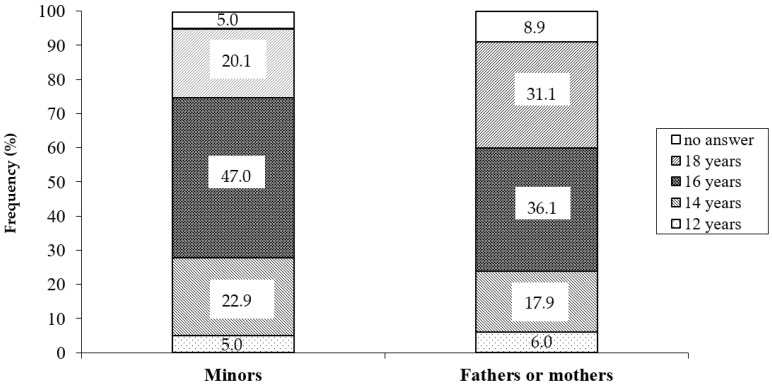
Answers of minor and parents about the age at which they believe that minors are competent to make decisions about their health.

Children and parents were asked about the age at which they believe that the minors are competent to make decisions about their health (Figure 2). Of the minors, 20.1% answered 18 years, 47.0% answered 16 years, 22.9% answered 14 years, and 5.0% answered 12 years. In the same question, 31.1% of parents think that minors should not make decisions about their health until they reach 18 years, 36.1% answered 16 years, 17.9% answered 14 years, and 6.0% thought 12 years was sufficient. There were statistically significant differences between minors and parents in relation to subjects who answered 18 years (20.1% vs. 31.1%; *p* = 0.003) and 16 years (47.0% vs. 36.1%; *p* = 0.008). Thus, adding all the answers, 74.9% of the minors think that at 16 years of age the minor is competent to make decisions about their health, and in the case of parents, this percentage is 60.0%. 

In the group of minors, we have compared these responses based on the age of the respondent, establishing two groups, subjects aged 16 and 17, and those under 16 years of age, and we found statistically significant differences (*p* = 0.000). Thus, in the group of minors between 16 and 17 years, 91.8% thought that minors can make decisions from the age of 16, while this percentage drops to 52.2% when the group of minors was aged less than 16. 

Minors were asked questions about decisions in the case of refusal of treatment. A total of 86.7% of minors believe they are competent to give an opinion on the acceptance or rejection of the proposed surgical intervention. This percentage drops to 65.8% when they respond if they are prepared to decide for themselves (without parental intervention) whether to accept or reject surgery. When minors were asked if their decision should be respected if they refuse surgery against the advice of the doctor and the wishes of the parents, 60.9% answered affirmatively. A total of 39.1% of parents believe that the minor’s decision should be respected if he refuses surgery.

### 3.4. Age Maturity to Decide in Medical Situations of Different Complexity and Severity

We ask minors and their parents about the age at which they believe a minor is competent to consent in various medical situations. We have included generic situations, which serve as examples, and which we consider to be easy to understand. We have chosen the following: invasive cardiac surgery as an example of serious surgical intervention, cosmetic surgery as an example of treatment demanded by adolescents and often questioned by parents, blood transfusion as a medical action that generates a great ethical and legal debate when it is rejected for religious reasons, and extraction of blood for analysis as an example of light medical procedure. In medical practice, informed consent is not usually obtained in blood extractions. The results obtained are shown in Table 3 and Table 4.

In the case of invasive cardiac surgery, 71.0% of minors and 71.8% of parents thought that the patient should be at least 18 years old to consent. As regards cosmetic surgery, 77.1% of minors and 85.1% of parents considered that the patient should be at least 18 years old to consent (Table 3). We have found statistically significant differences in the answers obtained between minors and parents. Additionally, we found statistically significant differences between the responses of the minors and the sex of the minor (*p* = 0.049). Of the 161 girls in our sample, only 2 (1.24%) considered that the age to decide on this type of intervention should be 18 years, the rest (98.76%) answered that it should be 16 years. However, in males, 13.5% answered that it should be at 18 years of age, and 86.5% at 16 years of age. 

In the case of blood transfusion, 38.1% and 25.8% of minors thought that the patient must be at least 18 years old and 16 years old to consent, respectively. In the case of parents, the percentages are 53.0% and 14.0%, respectively. In both cases, we found statistically significant differences between the responses from the minors and from the parents (*p* = 0.000). Regarding the extraction of blood for analysis, 55.0% of minors and 34.1% of parents thought that 12 years was sufficient to make decisions (*p* = 0.000) (Table 4). 

When the kappa index was applied to evaluate the degree of agreement among the replies of children and parents, statistical significance was obtained for the opinions concerning cosmetic surgery (k = 0.326; *p* = 0.001) and blood transfusions (k = 0.206; *p* = 0.048), but not in the case of a severe surgical intervention (k = −0.077; *p* = 0.462) or for the extraction of blood for analysis (k = 0.077; *p* = 0.377).

## 4. Discussion

Obtaining informed consent from the healthcare professional aims to ensure that patients have enough information to make a voluntary decision about their care [16]. Doctors have a great responsibility because they have to determine the competence of children to give their consent under the conditions stated by law, and sometimes some difficulties become problematic concerning the abilities of pediatric patients to make well-considered decisions [3]. Some empirical research on children’s competence to consent has been conducted using the MacCat-Treatment (MacCAT-T), but none of these studies tested the reliability and validity of the structured assessment instrument against a reference standard [17]. There are several reasons why assessing the capacity of adolescents is challenging, and makes setting age limits for health decisions very complex. 

Hein et al. [18] demonstrated that children’s decision-making competence regarding clinical research could be assessed using an instrument, the modified MacArthur Competence Assessment Tool for Clinical Research (MacCAT-CR), concluding that generally, children older than 11.2 years may be competent to consent. The MacCAT-CR is a semistructured interview format which measures the four aspects of decision-making capacities that reflect competence standards in most jurisdictions (understanding, appreciation, reasoning, and expressing a choice), estimating a cutoff score, above which competence is likely. 

In our study, minors and parents were asked about the age at which they believe that the minors are competent to make decisions about their health: 20.1% of minors and 31.1% of parents believe that patients should not make decisions related to their health until they are 18 years old. Almost half of the minors (47.0%) responded that the appropriate age to make decisions is 16 years old, but if we add the responses obtained for previous ages, we can conclude that 74.9% of the minors surveyed consider that from 16 years of age, the minor is empowered to make decisions. In parents, this percentage is 60%. 

Competence is conditioned by the growth and maturation of the brain structure. The consideration of an adolescent as ‘mature’ or ‘immature’ from a neurological point of view is difficult because this transient developmental period is a dynamic and evolutionary process, and is highly variable from one individual to another, and there may also be fluctuations over time [19]. The teenage years represent a period of struggle between seeking independence from parents and continuing to depend on them for many basic needs. During this time, progressive and particular neurobiological changes occur in each subject. Simultaneous with these neurobiological changes, there are marked behavioral changes in risk-taking, judgment, and decision-making. On the other hand, the capacity to understand the consequences of a decision is highly dependent on the context and the situation, while cognitive and affective development is highly variable among adolescents [20]. Additionally, competence in children is related to life experience, such as personal experiences with the illness [21]. A patient’s competence is not necessarily constant, especially when minors are involved. The diversity of situations that may arise, and possible actions that might be taken complicate matters even further when estimating the capacity to make health decisions. The ability to understand, assess, reason, and express a decision taking into account the options according to one’s own values, and their potential consequences for a specific decision, so it can vary according to the action for which the decision is necessary. Competence is based on an autonomous decision oriented to a specific goal. Autonomy is a continuum, that is, it increases or decreases over time; however, the competence is dichotomous, for a specific decision, one is competent or not. 

In children, decision-making also depends on the relationship between the child, parents, and healthcare professionals, and varies depending on the disease state [22]. In many cases, in pediatric care, the therapeutic decision made exclusively by the minor can be illusory, and the parent’s point of view often influences the decision-making of children and adolescents. Unless clinicians perceive significant coercion, this situation is acceptable. In our results, 86.7% of the minors believed themselves competent enough to express an opinion on the acceptance or rejection of the proposed surgical intervention. However, when asked if they were willing to decide for themselves, the percentage dropped to 65.8%. Controversy is likely to revolve around the age at which children and young people are considered competent to refuse treatment based on their best interests and disagree with the views of healthcare professionals. Although it is logical to assume that if children are competent to consent to medical treatment, they are also competent to refuse it, there may be difficulties with this approach [3]. 

In our study, when analyzing the answers to make decisions about the age in the different situations raised, we observe that the age of 18 years is considered to be the most appropriate by a significant percentage of minors and parents, for interventions considered as severe and of risk (cardiac surgery), cosmetic surgery, and blood transfusions. In blood extractions, the age of 12 is considered sufficient by 55.0% of minors and 34.1% of parents. These results confirm that the evaluation of the minor’s competence should always be carried out to the specific health decision to be taken, and with the complexity it may entail. Thus, the fundamental element that modulates the degree of capacity is both the complexity and the consequence of the decision. 

There are situations where the competence to make medical-related decisions should not necessarily depend on age alone. For this reason, we think it more suitable to establish a subjective model related with the maturity of minors or, at least, a mixed model in which both age and maturity have a role. This agrees with the case Gillick v West Norfolk and Wisbech Health Authority (10 November–20 December 1984, date of decision) [8], in which it was established that basing the capacity to decide solely on age may be excessively rigid in certain cases. 

Parents are generally in a better position than others to understand their children’s needs and make appropriate decisions about their health care, knowing their children’s interests [23]. The primary care physician and nurse who cares for and follows the minor in their health problems, and the psychologist on certain occasions, also play an important role. In the case of noncompetent children, the parents have the responsibility to decide, although, in complex circumstances and situations where the child’s interests may be at risk, the law courts may be involved. 

One compelling reason for giving adults decision-making authority regarding the health care of minors is that their greater maturity and experience generally allows them to make more reliable judgments about what is best for the patient. However, as adolescents increase in maturity and experience, their competence to make decisions increases. The adults involved in making decisions about the minor’s treatment must take this maturity and experience into account, placing themselves in a context that can be called collaborative paternalism [24]. It is paternalistic in the sense that it assigns decision-making authority to adults, not only about treatment decisions, but also in specific cases when judgments must be made about the minor’s best interests. It is also collaborative to the extent that adolescents actively participate in and contribute to the decision-making process along with adults. The combination of collaborative paternalism and age as objective criteria can facilitate decision-making. It allows minors to progressively increase their role and participation as they gain more experience and develop greater maturity. 

The current study is not without limitations. Firstly, it is a study carried out by answering a questionnaire conducted through a personal interview, so the presence of the interviewer can exert an influence on the respondent. In addition, the questionnaire was applied to minors and parents one week after surgery. Future research would do well to collect data from prospective patients at the point of the first consultation or prior to surgery. Secondly, the minors have not been asked about the surgical intervention to which they have been subjected, but rather about generic and diverse situations that serve as examples. However, it allows comparable responses to be obtained among all study participants and to extrapolate the results obtained on the perception of minors and parents in interventions of varying severity. 

## 5. Conclusions

In the pediatric setting, competence is associated with cognitive ability, rationality, and age in a context of relationships based on trust, mutual respect, and communication between children, clinicians, and parents. Competence is based on an autonomous decision oriented towards a specific purpose, so each case and situation must be examined individually to determine if the minor meets the condition of maturity to decide. The ideal is to promote the minor’s participation in decision-making, giving them the opportunity to participate in the process in a manner appropriate to their capacity. 

## Figures and Tables

**Table 1:** Demographic characteristics of minors and parents.

	N	%
Sex of minor		
Male	142	47.1
Female	160	52.9
Minor’s age -mean (SD)- 15.0 years (1.8) Distribution of minor according to age		
12 or 13 years	73	24.2
14 or 15 years	81	26.8
16 or 17 years	148	49.0
History of previous interventions		
Yes	87	28.8
No	215	71.2
Severity of intervention		
Minor	167	55.3
Serious	135	44.7
Questionnaire answered by the father	42	13.9
Questionnaire answered by the mother	260	86.1
Parent’s age -mean (SD)- 43.1 years (6.3)		
Distribution of parents according to age		
30 to 39 years	78	25.8
40 to 49 years	181	59.9
50 to 59 years	39	12.9
60 or over	4	1.3

**Table 2:** Information provided by the doctor to the minor about the surgical procedure in relation to the severity of the intervention and age.

	N	%
Yes	249	82.5
Age		
Under 14 years old	42	16.9
Over 14 years old	207	83.1
Severity of intervention		
Minor	162	65.1
Serious	87	34.9
No	53	17.5
Age		
Under 14 years old	31	58.5
Over 14 years old	22	41.5
Severity of intervention		
Minor	5	9.4
Serious	48	90.6

**Table 3:** Replies to questions concerning age at which minors should be able to make decisions on a variety of medical conditions (invasive cardiac surgery and cosmetic surgery).

	Invasive Cardiac Surgery			Cosmetic Surgery		
	Minors N (%)	Father/ Mothers N (%)	*p*	Minors N (%)	Father/ Mothers N (%)	*p*
18 years	214 (71.0)	217 (71.8)	0.857	233 (77.1)	257 (85.1)	0.017 ^a^
16 years	54 (17.9)	45 (15.0)	0.379	42 (13.9)	12 (4.0)	0.000 ^b^
14 years	9 (3.0)	9 (3.0)	1	12 (4.0)	9 (3.0)	0.658
12 years	9 (3.0)	12 (4.0)	0.658	9 (3.0)	3 (1.0)	0.142
No answer	15 (5.0)	18 (6.0)	0.721	6 (2.0)	21 (6.9)	0.005

All comparisons were made analyzing the responses of the children to those of the parents. The *p* value was determined using Fisher’s exact test two-sided. *p* ≤ 0.05 was considered statistically significant. ^a^ OR 0.591; 95% CI: 0.390–0.896; ^b^ OR 3.904; 95% CI: 2.012–7.576.

**Table 4:** Replies to questions concerning age at which minors should be able to make decisions on a variety of medical conditions (blood transfusion and extraction of blood for analysis).

	Blood Transfusion			Extraction of Blood for Analysis		
	Minors N (%)	Father/ Mothers N (%)	*p*	Minors N (%)	Father/ Mothers N (%)	*p*
18 years	115 (38.1)	160 (53.0)	0.000 ^a^	15 (5.0)	78 (25.8)	0.000 ^d^
16 years	78 (25.8)	42 (14.0)	0.000 ^b^	45 (14.9)	56 (19.9)	0.276
14 years	54 (17.9)	45 (15.0)	0.379	67 (22.8)	36 (11.9)	0.001 ^e^
12 years	42 (13.9)	23 (10.0)	0.018 ^c^	166 (55.0)	103 (34.1)	0.000 ^f^
No answer	12 (4.0)	24 (8.0)	0.057	6 (2.0)	24 (7.9)	0.001

All comparisons were made analyzing the responses of the children to those of the parents. The *p* value was determined using Fisher’s exact test two-sided. *p* ≤ 0.05 was considered statistically significant. ^a^ OR 0.546; 95% CI: 0.345–0.755; ^b^ OR 2.156; 95% CI: 1.423–3.266; ^c^ OR 1.960; 95% CI: 1.147–3.348; ^d^ OR 0.150; 95% CI: 0.084–0.268; ^e^ OR 2.107; 95% CI: 1.355–3.276; ^f^ OR 2.358; 95% CI: 1.698–3.276.

## Data Availability

All data generated and analyzed for the current study are included in this article.
